# High genotypic diversity and a novel variant of human cytomegalovirus revealed by combined UL33/UL55 genotyping with broad-range PCR

**DOI:** 10.1186/1743-422X-6-210

**Published:** 2009-11-26

**Authors:** Merlin Deckers, Jörg Hofmann, Karl-Anton Kreuzer, Henrike Reinhard, Abigail Edubio, Hartmut Hengel, Sebastian Voigt, Bernhard Ehlers

**Affiliations:** 1 P14 Molekulare Genetik und Epidemiologie von Herpesviren, Robert Koch-Institut, Nordufer 20, 13353 Berlin, Germany; 2 Institut für Medizinische Virologie, Charitéplatz 1, 10117 Berlin, Germany; 3 Klinik I für Innere Medizin, Joseph-Stelzmann-Straße 9, 50924 Köln, Germany; 4 Institut für Virologie, Heinrich-Heine-Universität Düsseldorf, Moorenstrasse 5, 40225 Düsseldorf, Germany; 5 P11 HIV-Variabilität und molekulare Epidemiologie, Robert Koch-Institut, Nordufer 20, 13353 Berlin, Germany; 6 FG12 Virale Infektionen, Robert Koch-Institut, Nordufer 20, 13353 Berlin, Germany

## Abstract

The known strains of human cytomegalovirus (HCMV) represent genotypic variants of a single species, and HCMV genotypic variability has been studied in order to reveal correlations between different disease patterns and the presence of certain HCMV genotypes, either as single or as multiple infections. The methods used for the detection of HCMV genotypes have not always been sophisticated enough to achieve complete comprehensiveness, mainly because only one genotype is usually detected in a certain specimen, due to primer specificity and genome copy number. To improve detection of variant HCMV genotypes in mixed infections, we developed PCR assays with degenerate primers targeting two variable HCMV genes, glycoprotein B (gB, *UL55*) and the G-protein-coupled receptor gene *UL33*. Primers were designed to bind conserved sites in the genomes of HCMV variants and great ape CMVs. To analyse if samples contained one or more HCMV genotypic variants, PCR assays were supplemented with oligonucleotides containing locked nucleic acids. This broad-range PCR methodology and subsequent sequence analysis detected all gB/*UL55 *and *UL33 *genotypic variants known to date in primary clinical specimens, but also revealed that many samples contained genotype mixtures. Importantly, a novel *UL33 *genotypic variant could be discovered in several specimens, and one HCMV isolate was plaque-purified containing the novel *UL33 *genotype and a so far undescribed variant of gB.

## Introduction

The existence of genotypic variants of human cytomegalovirus (HCMV) was first reported by Chou & Dennison [1] by analysing glycoprotein B (gB) gene sequences spanning the gB cleavage site (CLS) of different HCMV isolates. Four distinct CLS genotypes (gB_CLS_-1 to -4) were reported. Later it was shown that gB exhibits variability also at the N-terminus (gB_N_-1 to -4) as well as at the C-terminus (gB_C_-1 to -2) [2]. Further studies provided evidence for homologous recombination between gB_CLS_, gB_N _and gB_C _genotypes [3]. A fifth gB_CLS _genotype was detected in several AIDS patients [4]. More recently, additional distinct gB_N _sequences were described in AIDS patients, but not assigned to a genotype numbering system [5,6]. Several laboratories investigated HCMV genotype variability using PCR-based methods to unravel possible relationships between certain HCMV CLS genotypes and disease outcome, and although results have been conflicting at times, the occurrence of infections with more than one HCMV genotype and its impact on severity of HCMV disease has been considered crucial [reviewed in [7-14]].

For studies of these types, a comprehensive detection of all HCMV variants is highly desirable. In principle, this can be done with a set of independent and specific PCR assays, e.g., specific multiplex PCR, by restriction length polymorphism analysis, or by microarrays [1,15-17]. However, sequences of unknown HCMV variants would remain undetected. This is a serious drawback, in particular because recent discoveries of non-human CMV in both chimpanzees and gorillas revealed a considerably higher diversity than in humans [18].

An unprejudiced way of analysing specimens for the presence of known and unknown herpesviruses is to perform PCR with degenerate primers targeting highly conserved sites within the herpesvirus genome. Such assays proved to be highly effective in the discovery of herpesviruses [reviewed in [19]]. However, in mixed herpesvirus infections, usually only one viral genome is preferentially amplified, due to genome copy number or preferential primer binding. Using oligonucleotides substituted with locked nucleic acids (LNA), we recently developed a variant of the universal herpesvirus PCR and applied it to the detection of unknown herpesviruses in Old World monkeys. Up to three different monkey herpesviruses could be amplified from single specimens, thereby revealing the presence of hitherto unknown herpesviruses [20].

The aim of the present study was to universally determine variable genotypes of two HCMV genes, gB (*UL55*) and *UL33*. The latter encodes a G-protein-coupled receptor (GPCR) and is present in human and animal CMVs. *UL33 *signals constitutively, interferes with cellular signaling networks and modulates cellular function after infection, thereby probably contributing to HCMV-associated pathology [[21,22]; reviewed in [23]]. *UL33 *exhibits sequence variability but this information came from a limited number of strains [24]. Here we studied whether variable genotypes of HCMV gB and the *UL33*-encoded GPCR both can be universally and differentially identified in human clinical specimens by degenerate and LNA-substituted PCR.

## Methods

### Sample collection, cell culture, and DNA preparation

Twenty-eight blood samples pre-tested positive for HCMV with a pan-herpes PCR assay [25] were selected from a random collection of bone marrow transplanted, HCMV-seropositive children and from patients suffering from HIV infection or from different haematologic diseases. DNA was extracted by standard methods.

In addition, a collection of 89 HCMV patient isolates was analyzed after virus expansion on human MRC-5 fibroblasts (ATCC CCL-171, passages 3-15) upon HCMV isolation from various clinical specimens (throat washing, urine, EDTA blood, bronchoalveolar lavage fluid) which were simultaneously tested to be HCMV DNA-positive by routine PCR detecting HCMV polymerase. DNA was extracted from the pellets of infected cells after the second to sixth-passage. Plaque purification of HCMV isolates was performed using MRC-5 cells by limiting dilution of supernatants of infected cultures in log2 steps in a 96-well plate format. Wells containing a single plaque were harvested 7 days post infection followed by a second plaque purification using 48-well plates. HCMV plaques were then propagated in a 6-well plate the cells of which were used for DNA preparation by standard methods.

### Consensus-PCR assays for the amplification of genotypic variants of glycoprotein B (*UL55*)

For the comprehensive amplification of the CLS of HCMV gB genes, degenerate and deoxyinosine-substituted (deg/dI) primers were deduced from the gB/*UL55 *gene of HCMV strain AD169 in a nested format (primer set CMV-gBp1; Table 1). Binding sites were selected on the basis of a multiple alignment of all human and primate CMV gB genes available in Genbank. Primers were degenerated and substituted with deoxyinosine at their 3'-end. PCR was performed with up to 12.3 μl of DNA, containing a maximum of 250 ng DNA, in first round amplification. PCR mix (12,7 μl; containing 1× AmpliTaq™ buffer, 2,0 mM MgCl_2_, 2-5% DMSO, the four dNTPs at 200 mM each, 2.0 units of AmpliTaq Gold Polymerase and 1 μM of each sense- and antisense primer) and - if necessary - water was added to achieve a total volume of 25 μl. For second round amplification, 1 μl of the first-round amplification product was added. Thermal cycling was performed in thermocyclers of type TgradientS (Biometra, Germany), under the following conditions: Initial denaturation 95°C for 2 min, 40 cycles with denaturation at 95°C for 30 sec, annealing at 46°C for 30 sec and elongation at 72°C for 1 min; final elongation at 72°C for 10 min. The annealing-to-elongation phase (ramp-time) was prolonged to 0,09°C/min. The PCR reactions were performed in duplicate.

**Table 1:** Primers for amplification of the glycoprotein B and UL33 genes

Primer-set	Name of primer	PCR round	Sequence 5'-3'	Product length^$^
CMV-gBp1	4007s	1	GGTGACGTGGTTGACAT(n/i)TC(n/i)CC	800
	4007as		GCAATCGGTTTGTTGTA(n/i)AT(n/i)GC	
	4125s	2	GCGGACTCGGTGAT(b/i)TC(n/i)TGGGA	550
	4125as		GCTGACGGGTTGATCTTGCT(r/i)AG	
CMV-gBp2	4524s	1	GGAATCCAGGAT(y/i)TGGT(r/i)CCT	1790
	4524as		TGTCCTCACCCAG(y/i)TG(n/i)CC(r/i)TA	
	4525s	2	CTGGTGCCTGG(y/i)AG(y/i)CTGCG	1590
	4525as		CGGTTTGTTGTA(r/i)ATGGC(n/i)GA	
CMV-UL33	4068s	1	GGAAACGCAGCTC(n/i)CG(r/i)TA(r/i)TA	2000
	4068as		TGTCCTGGTGCTCGCA(r/i)TA(r/i)TG	
	4069s	2	GCGCCGCTG(n/i)AG(n/i)CC(r/i)AT	1700
	4069as		GGAGAAGTC(y/i)CGGGT(r/i)GT(n/i)AT	

^# ^I = Inosine

^$^bp (approximate values)

A second nested deg/dI primer set was selected (primer set CMV-gBp2; Table 1) which amplifies two thirds of the CMV gB/*UL55 *gene comprising the N-terminal variable region and the CLS of gB. PCR was performed with the TaKaRa-Ex PCR system (Takara Bio Inc., Japan) using the following protocol: The reaction mixture was set up on ice and contained 50 μL of ExTaq buffer with MgCl_2_, 400 μM of each dNTP, 400 nM of each primer, 2.5 units of TaKaRa ExTaq polymerase (TaKaRa Bio Inc, Japan). Thirty cycles of amplification were performed, each cycle consisting of a denaturing step at 98°C for 20 sec, an annealing step at 45°C for 30 sec and an elongation step at 72°C. The duration of the elongation step in the first 15 cycles was 7 minutes and in the last 15 cycles 10 min +5 sec ramp time for each cycle. The final elongation step was performed for 30 minutes at 72°C. The PCR reactions were performed in duplicate.

**Figure 1:**
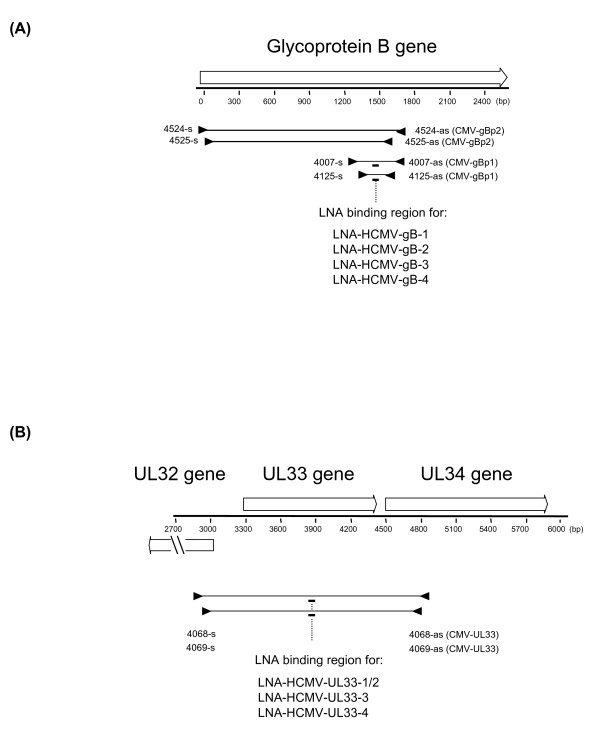
**Amplification of HCMV gB/*UL55 *and *UL33 *using LNA-supplemented consensus PCR**. Schematic diagram of the analysis strategy. Nested PCR is performed with deg/dI primers (black triangles). The primers CMV-gBp1 target the CLS of the gB gene, and the primers CMV-gBp2 target the gB gene from the N-terminus to the CLS **(A)**. The complete *UL33 *gene is amplified by the CMV-UL33 primers **(B)**. The binding regions of the LNA-oligonucleotides are present in the amplified sequences of both the first and the second PCR round, and represented by short thick lines (the position varies slightly for each LNA). The targeted HCMV genotypes are indicated.

The primers of the second round of CMV-gBp1 and CMV-gBp2 amplified fragments of approximately 550 bp and 1590 bp, respectively. Binding regions of all gB primers are depicted in Figure 1.

### Consensus-PCR assay for the amplification of genotypic variants of *UL33*

For the broad amplification of full-length HCMV *UL33 *genes (plus short, flanking regions of UL32 and UL34 sequences), nested deg/dI sense primers were deduced from the UL32 gene and antisense-primers from the *UL34 *gene of HCMV strain AD169 (primer set CMV-*UL33*; Table 1). Primer binding sites were selected on the basis of multiple alignments which comprised all *UL32 *and *UL34 *genes, respectively, of human and primate CMV available in Genbank. Primers were degenerated and substituted with deoxyinosine at their 3'-end. Primers used in the second round amplified fragments of approximately 1700 bp, and primer binding regions are depicted in Figure 1. PCR was performed with the TaKaRa-Ex PCR system as described above. The PCR reactions were performed in duplicate.

### PCR for specific amplification of the *UL33-5 *gene

*UL33*-5 sequences were amplified with the primer set 4347-s (5'-CGTTCAACTTACAT AGTTTTGACG-3') and 4347-as (5'-GCGTCTGTAGCGCCACAAAAG-3') at 61°C annealing temperature. Amplified products had a size of 346 bp.

### Design of LNA-oligonucleotides specific for gB/*UL55 *and *UL33 *genes

**Figure 2:**
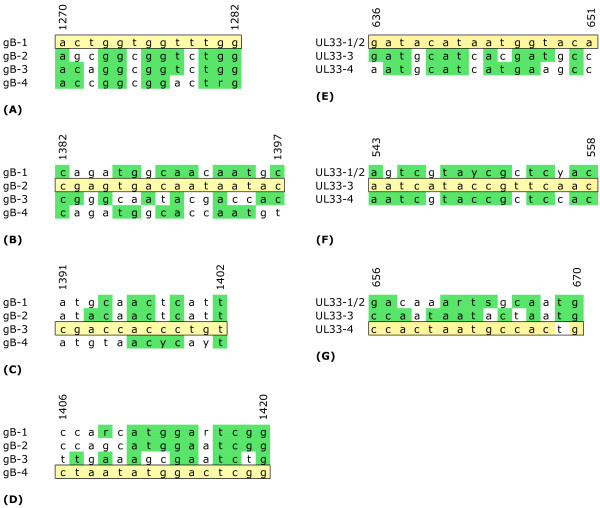
**Binding sites of LNA-oligonucleotides in the gB/*UL55 *and *UL33 *genes of HCMV**. The binding sites of the LNA used in this study for specific inhibition of HCMV gB/ *UL55 ***(A-D) **and *UL33 ***(E-G) **genotype amplification are boxed and aligned with the corresponding regions of the other three gB genotypes. The LNA- bases and sequences identical to the LNA sequence are highlighted in yellow/green. On top of alignments A-D, the positions in the gB genes of gB genotype CLS-1 [M60929] **(A)**, gB genotype CLS-2 (strain AD169)**(B)**, gB genotype CLS-3 [M85228]**(C) **and gB genotype CLS-4 [M60926] **(D) **are shown. LNA-gB-1 and LNA-gB-4 bind to the sense strand, LNA-gB-2 and LNA-gB-3 bind to the antisense strand. On top of the alignments E-G, the positions in the genes of UL33 genotype 1 (strain Merlin) **(E)**, UL33 genotype 3 (strain Toledo)**(F) **and UL33 genotype 4 (strain AD169)**(G) **are shown. LNA-UL33-1/2 binds to the antisense strand, LNA-UL33-3 and LNA-UL33-4 to the sense strand.

Four LNA-oligonucleotides (TIB MOLBIOL GmbH, Berlin, Germany) were designed to specifically inhibit the amplification of one of the HCMV gB genotypes 1 to 4. Their binding sites were located in the region of the CLS. The four HCMV *UL33 *genotypes were targeted by only three LNA-oligonucleotides (LNA-HCMV-UL33-1/2, -3 and -4) because for the closely related genotypes *UL33*-1 and -2, selection of discriminating LNA-oligonucleotides was not possible. Therefore, the common LNA-oligonucleotide UL33-1/2 was used (Figures 1 and 2; Table 2).

**Table 2:** LNA sequences

LNA (name)	LNA (Sequence)^$^	Target DPOL sequence	T_m _of DNA-oligomer^#^	T_m _of LNA-oligomer^#^
LNA-HCMV-gB-1	5'-+c+c+a+a+a+c+c+a+c+c+a+g+t+g-NH_2_	HCMV gB-1	50	87
LNA-HCMV-gB-2	5'-+c+g+a+g+t+g+a+c+a+a+t+a+a+t+a+c-NH_2_	HCMV gB-2	45	80
LNA-HCMV-gB-3	5'-+c+g+a+c+c+a+c+c+c+t+g+t-NH2	HCMV gB-3	49	86
LNA-HCMV-gB-4	5'-+c+c+g+a+g+t+c+c+a+t+a+t+t+a+g-NH_2_	HCMV gB-4	45	85
				
LNA-HCMV-UL33-1/2	5'-+t+g+t+a+c+c+a+t+t+a+t+g+t+a+t+c-NH_2_	HCMV UL33-1/2	42	79
LNA-HCMV-UL33-3	5'-+a+a+t+c+a+t+a+c+c+g+t+t+c+a+a+c-NH_2_	HCMV UL33-3	47	80
LNA-HCMV-UL33-4	5'-+c+c+a+c+t+a+at+g+c+c+a+ct+g-NH_2_	HCMV UL33-4	50	80

^$^All bases in LNA conformation are preceeded by +

^#^T_m _= melting point; calculated with the algorithm provided on the website http://lna-tm.com/

### Sequence analysis and phylogenetic tree construction

PCR product purification, direct sequencing with dye terminator chemistry, as well as nucleotide and amino acid sequence analysis were performed as described [26]. Sequencing reactions were performed on both strands with a total redundancy of at least four-fold. Sequence chromatograms were assembled with the Seqman module of the Lasergene software (GATC, Konstanz, Germany). BLAST searches were performed using the NCBI database. ORF prediction, calculation of identity values and multiple sequence alignments (ClustalW) were performed using MacVector software (Version 10.0). For phylogenetic tree construction, a nucleotide sequence alignment was analysed with the neighbor-joining method (MacVector). Alternatively, the Proml module of the PHYLIP program package on the Trex server http://www.trex.uqam.ca was used for Maximum-Likelihood analysis.

### Numerical designation of genotypes and nucleotide sequence accession numbers

**Figure 3:**
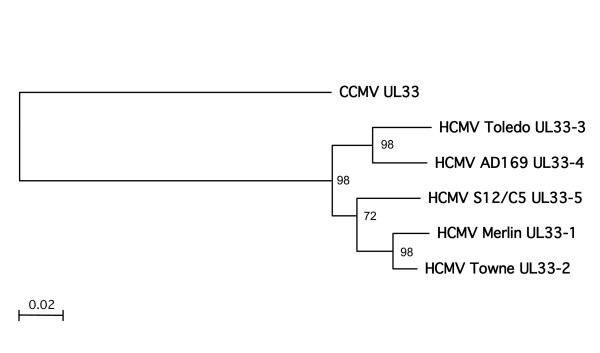
**Phylogenetic analysis of *UL33 *genotypes**. UL33 proteins (Figure 4) were aligned to construct a phylogenetic tree using the Neighbor-joining method. A rooted phylogram is shown, with the *UL33 *of the chimpanzee cytomegalovirus CCMV as outlier. The branch length is proportional to evolutionary distance (scale bar). Results of bootstrap analysis (1000 replicates) are indicated at the nodes of the tree.

The gB sequences of published HCMV strains were assigned to gB_CLS _genotype numbers 1-4 according to the numbering in earlier studies [1,4]: Merlin [GenBank acc.-no. NC_006273], gB_CLS _genotype 1; Towne [AY315197], gB_CLS _genotype 1; AD169 [NC_001347], gB_CLS _genotype 2; Toledo [AC146905], gB_CLS _genotype 3; and isolate C194A [M60926] gB_CLS _genotype 4. The complete gB *UL55 *sequence of the HCMV S3 isolate was determined here for comparative purposes [GU180092]. The acc.-no. for chimpanzee cytomegalovirus (CCMV) UL-33 shown in figure 3 is [NC_003521].

We did not find a numbering system for *UL33 *genotypes in the literature, but we observed by phylogenetic tree construction that the published HCMV *UL33 *genes clearly branch in four clades (not shown). Therefore, we assigned the *UL33 *genotypes to numbers 1-4 for the purpose of this study (Figure 3). The novel *UL33 *genotype, presented here, was tentatively named *UL33-5*. The gB *UL55 *and *UL33 *sequences of the novel HCMV variant (HCMV-C5) were deposited in GenBank under the acc.-nos. [GU180093] and [GU180094], respectively.

## Results

### Universal screening of human blood for the presence of variant HCMV gB/*UL55 *genotypes

In order to test the ability of the CMV-gBp1 PCR to universally detect all known CLS genotypes of HCMV gB, a random collection of 28 HCMV-positive clinical blood specimens (S1-S28) was tested with the CMV-gBp1 PCR (without LNA addition). Amplimers were sequenced and the genotype determined. All known gB_CLS _genotypes (types 1 to 5) were detected. The genotype gB_CLS_-1 was detected in 13/28, gB_CLS_-2 in 2/28, gB_CLS_-3 in 10/28, gB_CLS_-4 in 2/28 and gB_CLS_-5 in 1/28 specimens (Table 3). The sequences differed only slightly from the published sequences or were identical (data not shown).

**Table 3:** LNA-based detection of HCMV genotypic variants of glycoprotein B and UL33 GPCR

	gB_CLS _types (gBp1 PCR)		UL33 types (CMV-UL33 PCR)	
**Specimen number**	**without LNA**	**with LNA**	**without LNA**	**with LNA**

S1^§^	2	3, 5^#^	3	4
S2	2	3	3	-
S3	5	-	1	4
S4	4	1	3	-
S5	1	-	4	-
S6	1	2	2	-
S7	1	-	2	-
S8	3	-	2	-
S9	1	3	2	-
S10	3	-	2	-
S11	3	1	2	-
S12	3	-	1	5
S13	1	-	1	-
S14	3	-	4	-
S15	3	-	4	-
S16	3	-	1	-
S17	1	-	2	-
S18	1	-	4	-
S19	3	-	4	-
S20	1	3	1	4
S21	1	-	2	-
S22	4	3	1	-
S23	1	-	1	-
S24	3	-	1	-
S25	1	-	2	-
S26	1	-	2	-
S27	1	-	2	-
S28	3	4	1	4

C1	1		2	
C2	3		2	
C3	3		1	
C4	1		1	
C5	2		5	

^# ^Genotype 5 was detected after addition of two LNAs (LNA-HCMV-gB-2 and LNA-HCMV-gB-3)

The existence of mixed infections with different HCMV variants was analysed by repeating the PCR in the presence of an LNA-oligonucleotide perfectly matching the gB genotype detected in the first experiment. Prior to use of these LNA-oligonucleotides, we confirmed that they specifically inhibited the amplification of a perfectly matching gB genotype in the CMV-gBp1 PCR (not shown). In additional pre-tests, we achieved selective amplification of a single CLS genotype from mixtures of strains, representing four CLS genotypes, using CMV-gBp1 PCR and up to three LNA oligonucleotides. These pre-tests were carried out in the same manner as reported by Prepens *et al. *[20], and are not described in detail.

In the presence of the genotype-specific LNA-oligonucleotides, an amplimer was obtained by CMV-gBp1 PCR from 9/28 HCMV-positive blood specimens. Sequence analysis revealed the presence of a second CLS genotype in each of the nine specimens. The PCR assay was repeated again, this time in the presence of two LNA-oligonucleotides in each reaction mixture. In one sample, a third CLS genotype was found. Thus, upon addition of the respective LNA, gB_CLS_-2 and -3 were detected in initially gB_CLS _-1-positive specimens, gB_CLS_-3 and/or gB_CLS_-5 in gB_CLS_-2-positive specimens, gB_CLS_-1 or gB_CLS_-4 in gB_CLS_-3-positive specimens and gB_CLS_-1 or gB_CLS_-3 in gB_CLS_-4-positive specimens (Table 3). In summary, LNA supplementation of the PCR enabled the differentiation of a total of 38 CLS sequences, ten more than with degenerate PCR alone.

### Universal screening of human blood samples for the presence of HCMV *UL33 *genotypes

The same set of 28 primary specimens was tested for the presence of different *UL33 *genotypes with the CMV-UL33 primer set and - in subsequent experiments - in the presence of *UL33*-type-specific LNA-oligonucleotides. Single *UL33 *genotypes were detected in 23 samples, and 2 genotypes in five samples. All known *UL33 *genotypes known to date (types 1 to 4; Table 3) were detected. Without LNA addition, *UL33*-1, *UL33*-2, *UL33*-3 and *UL33*-4 were detected in 9, 11, 3 and 5 samples, respectively. The sequences differed only slightly from the published sequences or were identical (data not shown). Upon addition of the respective LNA, *UL33*-4 was detected in initially *UL33*-1-positive samples and *UL33*-1 in *UL33*-3-positive samples. Most importantly, a hitherto unknown HCMV *UL33 *genotype was discovered: from a primary blood specimen (S12), genotype 1 was initially amplified, but after addition of LNA-UL33-1 to the PCR reaction the novel *UL33 *sequence was detected (Table 3). It was tentatively named HCMV *UL33 *genotype 5 (*UL33*-5) (Table 3).

In pairwise sequence comparisons, *UL33*-5 was the most distant genotype, compared to the other four genotypes. It was most closely related to *UL33*-2 (88% at the nucleic acid level and 93% at the aa level). In phylogenetic analysis using the Neighbor-joining method, the *UL33*-5 genotype formed a branch with *UL33*-1 and -2 (Figure 3). In Maximum-Likelihood analysis, the same tree toplogy was obtained (not shown).

**Figure 4:**
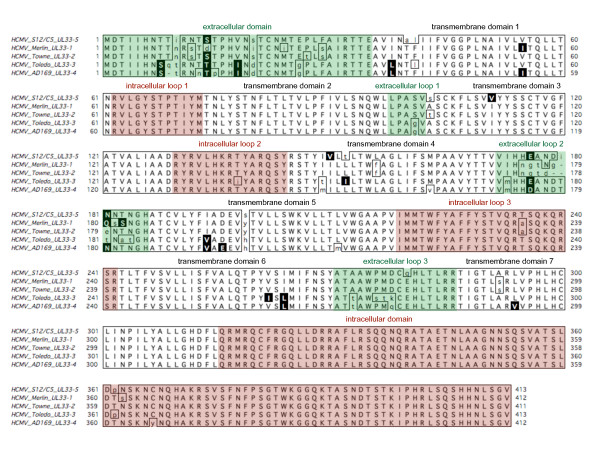
**Multiple alignment of HCMV *UL33 *genotypes**. Amino acid sequences were deduced from the complete, spliced HCMV UL33-1 to UL33-5 ORFs. An alignment of the complete HCMV UL33 proteins was performed using the ClustalW module of MacVector version 10.6.0. Identical and similar amino acids are boxed and the latter additionally inversed. Mismatches are shown in lower case. The structural domains of the UL33 GPCR were determined as described in the *Results *section, and depicted in white (transmembrane domains), green (extracellular domains and loops) and red (intracellular domains and loops).

Since *UL33 *has been reported to be a spliced gene with two exons [21], we inspected the *UL33*-5 ORF for the presence of splice sites. In sample S12, a contiguous consensus sequence of 1715 bp spanning the 5'-end of the inversely oriented UL32, the complete *UL33*-5 ORF and the 5'-end of UL34 identified strong and conserved splice donor and acceptor signals (not shown). The spliced *UL33*-5 gene was determined to be 1242 bp in length (Exon 1: 27 bp; exon 2: 1212 bp) with a coding capacity of 413 amino acids. Based on a multiple amino acid alignment of *UL33 *proteins, putative structural receptor domains are shown in Figure 4.

### Isolation and plaque-purification of a virus containing the novel genotype *UL33-5*

**Figure 5:**
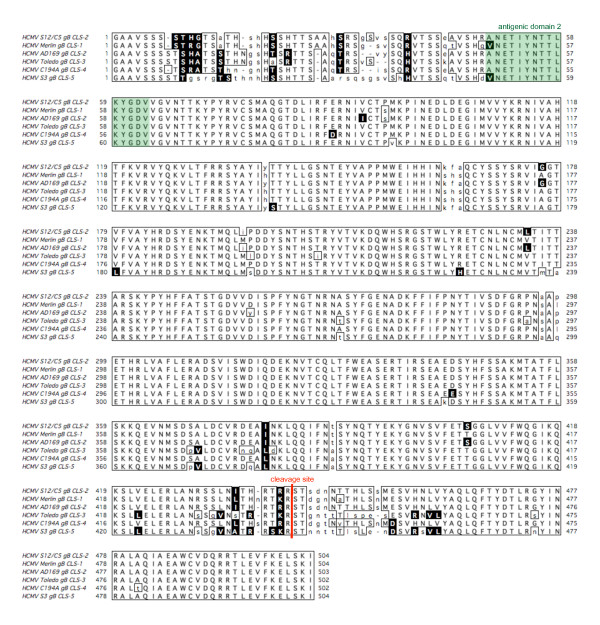
**Multiple alignment of HCMV gB/*UL55 *variants**. An alignment of HCMV gB amino acid sequences was performed as described in the legend to Figure 4. The alignment starts with Gly^20 ^of the gB protein (the first 19 residues were not determined because their coding sequence is not part of the amplified gB fragment). The core region of the antigenic domain 2 [29] and the CLS are marked in green and red, respectively.

Next, we wanted to isolate HCMV carrying the novel *UL33*-5 genotype. Since the clinical specimen S12 was not suitable for cell culture experiments, we collected eighty-nine additional specimens which had been tested positive for HCMV in routine diagnostics, and cultured them on MRC-5 cells. Early-passage cultures were analysed with the primer set 4347 which specifically detects *UL33*-5 but not *UL33*-1 to *UL33*-4. Fifteen samples (17%) were positive. Since these cultures were also mixtures of strains (data not shown), we re-tested five of them (C1-C5) with the degenerate primer set CMV-*UL33 *in order to identify a sample which predominantly contains a virus with the *UL33*-5 genotype. This was the case with culture C5. It was *UL33*-5-positive in both PCR assays and therefore contained a virus with the *UL33*-5 genotype as a majority in the strain mixture (Table 3). HCMV-C5 was then plaque-purified by limiting dilution. After two rounds of plaque purification, seven plaques were isolated and passaged once. PCR followed by sequence analysis revealed that all contained *UL33*-5. To study the gB gene of this virus in more detail, we used two degenerate primer sets, CMV-gBp1 and CMV-gBp2. The latter amplified two thirds (roughly 1.6 kb) of the gB gene including both the N-terminus and the CLS. Sequence analysis revealed a CLS sequence similar to that of AD169 (gB_CLS_-2). However, it differed from AD169 at the N-terminus, and was identical to a divergent gB sequence detected by Steininger *et al. *in an AIDS patient [5]. Thus, we concluded that HCMV-C5 contains a so far undescribed gB_CLS_/gB_N _combination. Re-inspection of the *UL33*-1/*UL33*-5-positive specimen S12 with the CMV-gBp2 PCR revealed the same gB_CLS_/gB_N _combination (although in the previous experiment with CMV-gBp1 PCR gB_CLS_-3 was detected; Table 3). This indicated for S12 that this gB_CLS_/gB_N _combination might be part of HCMV genomes which contain either the *UL33*-1 or the *UL33*-5 genotype. A comparison of the HCMV-C5 gB gene with those of published strains is shown in Figure 5.

## Discussion

Here we present the application of a broad-spectrum technique for the universal detection and differentiation of variable genotypes of the gB and *UL33 *genes of HCMV. All known HCMV gB and *UL33 *genotypes could be amplified with two sets (CMV-gBp1, CMV-*UL33*) of universal primate CMV primers (Table 1). Most importantly, a novel *UL33 *genotype (*UL33*-5) was discovered that differs significantly from the known genotypes (Figure 3, 4). *UL33*-5 could be identified in a total of 16 clinical HCMV isolates, one of which was plaque-purified (HCMV-C5).

Both the isolate HCMV-C5 and the *UL33*-5-positive specimen S12 contained the same gB_CLS_/gB_N _combination. While gB_CLS _was similar to that found in AD169, gB_N _was not (Figure 5). An identical gB_N _sequence was detected previously in an HCMV isolate of an AIDS patient [5], however, the CLS sequence of that isolate was not analysed. The gB_CLS_/gB_N _found in HCMV-C5 (and the *UL33*-5-positive specimen S12) are reported here for the first time. During the preparation of this manuscript, two HCMV isolates were deposited in GenBank with almost identical genotype sequences for *UL33 *and *UL55 *(GQ396662 and GQ221975, respectively). It is inferred that the novel *UL33 *and gB sequences may be not rare.

The discovery of this novel HCMV variant indicates that the primer sets are of a sufficiently broad detection range. This is underscored by our recent discoveries of novel CMV gB and *UL33 *sequences in chimpanzees and gorillas where these primer sets were employed (CMV-gBp1: [18]; CMV-gBp2 and CMV-*UL33*: B. Ehlers, unpublished data). We infer from these findings that the universal primers would also detect unknown, even more aberrant HCMV genotypes and variants.

The differentiation of HCMV genotypes from mixed infections was achieved by performing PCR in the presence of LNA-oligonucleotides. Previously, LNA-supplemented PCR was used for the discovery and differentiation of non-human primate herpesviruses in multi-infected samples [20]. It was adapted here to differentiate HCMV genotypes, and proved to be equally effective. By this approach, single samples revealed up to three genotypes. The most important finding was the discovery of the novel *UL33*-5 genotype with LNA-supplemented consensus PCR in a clinical sample (S12) in which only the *UL33*-1 genotype was detected with consensus PCR alone (Tab. 3).

In principle, known HCMV genotypes can be amplified with specific primers. However, with more than ten N-terminal and CLS genotypes already known, this could become very laborious, making LNA-supplemented PCR approaches with degenerate primers as those presented here feasible alternatives. With this method, every genotype should be detectable, including unknown ones. In addition, for genotypes preferentially present in mixed infections or for the comprehensive analysis of sample material from rare patients, the LNA-based differentiation of genotypes from single samples is the method of choice.

Of note, the gB_CLS_-5 genotype has been detected in five HIV-positive patients [4]. In accordance with this, we detected gB_CLS_-5 in two HIV-positive patients (S1, S3; Table 3). At the N-terminus, the gB sequence of S3 was identical to gB_N _sequences which were retrieved from two HIV-infected patients [5] (since these authors did not analyse the CLS, further comparisons could not be done). These findings warrant further investigation on a possible association of HCMV carrying the gB_CLS_-5 genotype with HIV.

For a long time, gB has been in the focus as a suitable target antigen that might be utilized for vaccination and thus prevention of maternal cytomegalovirus infection. In a phase 2 placebo controlled, randomized and double-blind trial, a gB-based vaccine was found to have a vaccination efficacy of 50% [27]. Although the key epitopes for neutralizing antibodies are supposed to be conserved between the presently known gB genotypes [28], the core region of the antigenic determinant 2 [29] is not entirely conserved (Figure 5). HCMV variants may exist that are even more diverse and only insufficiently neutralized by the antibodies elicited by the gB vaccine. The broad-spectrum PCR methodology presented here may prove useful in detecting these variants.

Whether the GPCR protein encoded by *UL33 *has a natural ligand or functions merely through constitutive, i.e., ligand-independent intracellular signalling is not known. All published functional tests on HCMV *UL33 *were undertaken with the *UL33 *GPCR encoded by the laboratory strain AD169 [30,31]. When the five *UL33 *amino acid sequences were compared, intra-genotypic variation was most pronounced in certain sections. These correspond to the putative extracellular parts of the GPCR, whereas the intracellular parts show very low variability (Figure 4). Ligand interaction was attributed to the extracellular domain and extracellular loops (primarily loops 2 and 3) [32]. Since these domains revealed the highest variability in *UL33*, it may be useful to additionally examine *UL33 *genotypes other than AD169. This might help to identify a ligand for *UL33*.

Mixed infections with several herpesvirus genotypes are frequent, and especially immunocompromised patients are affected [8,13-19,33-35]]. In the latter situation, infections with multiple HCMV gB_CLS _genotypes correlate with an increased rate of graft rejection, higher viral loads, and frequency of infection with other herpesviruses [33,34,36,37]. For therapeutic purposes, it is therefore important to early identify genotypes that bear a higher virulence potential. Relationships between certain HCMV glycoprotein genotypes and organ manifestations or outcome of HCMV infections have been looked into. However, establishing correlations has frequently been difficult because of limited testing sensitivity with the majority of methods using a defined, narrow detection spectrum. Only two groups of investigators used partially degenerate primers [36,37]. Methods enabling the detection of multiple genotypes in a single sample were applied in two studies where approximately 25% to 29% of the samples were found to be multi-infected with HCMV strains of different gB genotypes [8,38]. In accordance with this, more than one HCMV gB genotype was detected in 32% of the samples S1-S28 in the present study. Thus, an improved technique for universally detecting and differentiating known and unknown genotypic HCMV variants in clinical specimens would enhance understanding multiple HCMV infections, the reasons for their occurrence, and the pathogenicity they cause. In contrast to detection systems used in earlier studies [1,8,36-39], the methodology presented here is the first that meets this requirement. It allows to comprehensively detect and differentiate cytomegaloviruses and might help to identify a correlation between a given genotype or a certain mixture of genotypes and disease.

## Competing interests

The authors declare that they have no competing interests.

## Authors' contributions

SV and BE designed research, MD and HR performed experiments, JH, AE, KAK and HH collected, pretested, and provided samples, and SV and BE wrote the manuscript. All authors read and approved the final manuscript.
